# Neurocognitive therapeutics: from concept to application in the treatment of negative attention bias

**DOI:** 10.1186/s13587-015-0016-y

**Published:** 2015-04-18

**Authors:** David M Schnyer, Christopher G Beevers, Megan T deBettencourt, Stephanie M Sherman, Jonathan D Cohen, Kenneth A Norman, Nicholas B Turk-Browne

**Affiliations:** Department of Psychology & Institute for Mental Health Research, University of Texas at Austin, Austin, TX 78712 USA; Princeton Neuroscience Institute, Princeton University, Princeton, NJ 08540-1010 USA; Department of Psychology, University of Texas at Austin, Austin, TX 78712 USA; Department of Psychology and Princeton Neuroscience Institute, Princeton University, Princeton, NJ 08540-1010 USA

**Keywords:** Attention bias, Real-time neurofeedback, Multivoxel pattern analysis (MVPA), Mood disorders, fMRI

## Abstract

There is growing interest in the use of neuroimaging for the direct treatment of mental illness. Here, we present a new framework for such treatment, neurocognitive therapeutics. What distinguishes neurocognitive therapeutics from prior approaches is the use of precise brain-decoding techniques within a real-time feedback system, in order to adapt treatment online and tailor feedback to individuals’ needs. We report an initial feasibility study that uses this framework to alter negative attention bias in a small number of patients experiencing significant mood symptoms. The results are consistent with the promise of neurocognitive therapeutics to improve mood symptoms and alter brain networks mediating attentional control. Future work should focus on optimizing the approach, validating its effectiveness, and expanding the scope of targeted disorders.

## Background

*Neurocognitive therapeutics* combines cognitive training with state-of-the-art neural-monitoring techniques in order to facilitate neuroplasticity. By combining behavioral paradigms with brain imaging, specific mental states of interest can be targeted directly and effectively. A particularly promising approach combines real-time functional magnetic resonance imaging (fMRI) with multivoxel pattern analysis (MVPA): a classifier can be trained to measure the presence of a mental state in brain activity patterns [1]; this measure can then be used to dynamically alter the behavioral paradigm, in essence adapting it to the personal ability of the individual. We have begun to apply this kind of approach in depressed adults with negatively biased attention, and our preliminary results are promising. The chief purpose of this article is to outline the methodological approach we have developed, rather than to report conclusive findings. Before doing so, however, we first describe some relevant prior work involving (1) behavioral-attention-training paradigms and (2) real-time fMRI neurofeedback.

### Behavioral attention training

The ability to control attentional capture and disengagement from affective stimuli is a crucial element of adaptive self-regulation [2]. For example, excessive attention to negative affective information has been identified as a fundamental process observed across diagnosis that may underlie the development of multiple disorders [3,4]. As a result, a number of investigators have developed and tested cognitive paradigms to train attentional control in an effort to diminish attentional bias to negative content. In prior work, we have shown that changes in attentional bias mediated the effect of attention training on depression symptom change [5,6]. Similar results have been found with depressed [7] and depression-vulnerable [8] individuals and in other psychiatric conditions [9-11], although null findings have also been reported [12]. One possible reason for the mixed results of prior attention-training work may be that it has involved delivering feedback based on behavior, and often without tailoring the feedback to the individual patient.

### Real-time fMRI neurofeedback

Real-time fMRI is an approach to brain imaging that involves simultaneously measuring and analyzing the blood-oxygen-level-dependent (BOLD) signal [13]. A number of researchers have used real-time fMRI to provide neurofeedback, by reflecting back to participants the results of the real-time analysis during the scanning session. Participants are encouraged to use this feedback and adjust their cognitive strategy to alter their neural response in real time [14]. Virtually all fMRI neurofeedback studies with clinical populations have used a block design approach in which participants are presented with visual feedback indicating the magnitude of the BOLD signal in a brain region of interest [15]. Frequently in such studies, the signal being measured cannot easily be tied directly to any particular mental state - it is often unclear what participants are actually doing. More recent applications have combined multiple brain-imaging techniques in an attempt to identify more specific mental states, such as positive emotion induction [16]. However, despite the multiple real-time brain measures (fMRI and EEG), the signals are not employed to directly alter a cognitive task. In particular, no real-time fMRI paradigm has targeted the negative attention bias in depression.

## Attention training with closed-loop real-time fMRI neurofeedback

**Figure 1:**
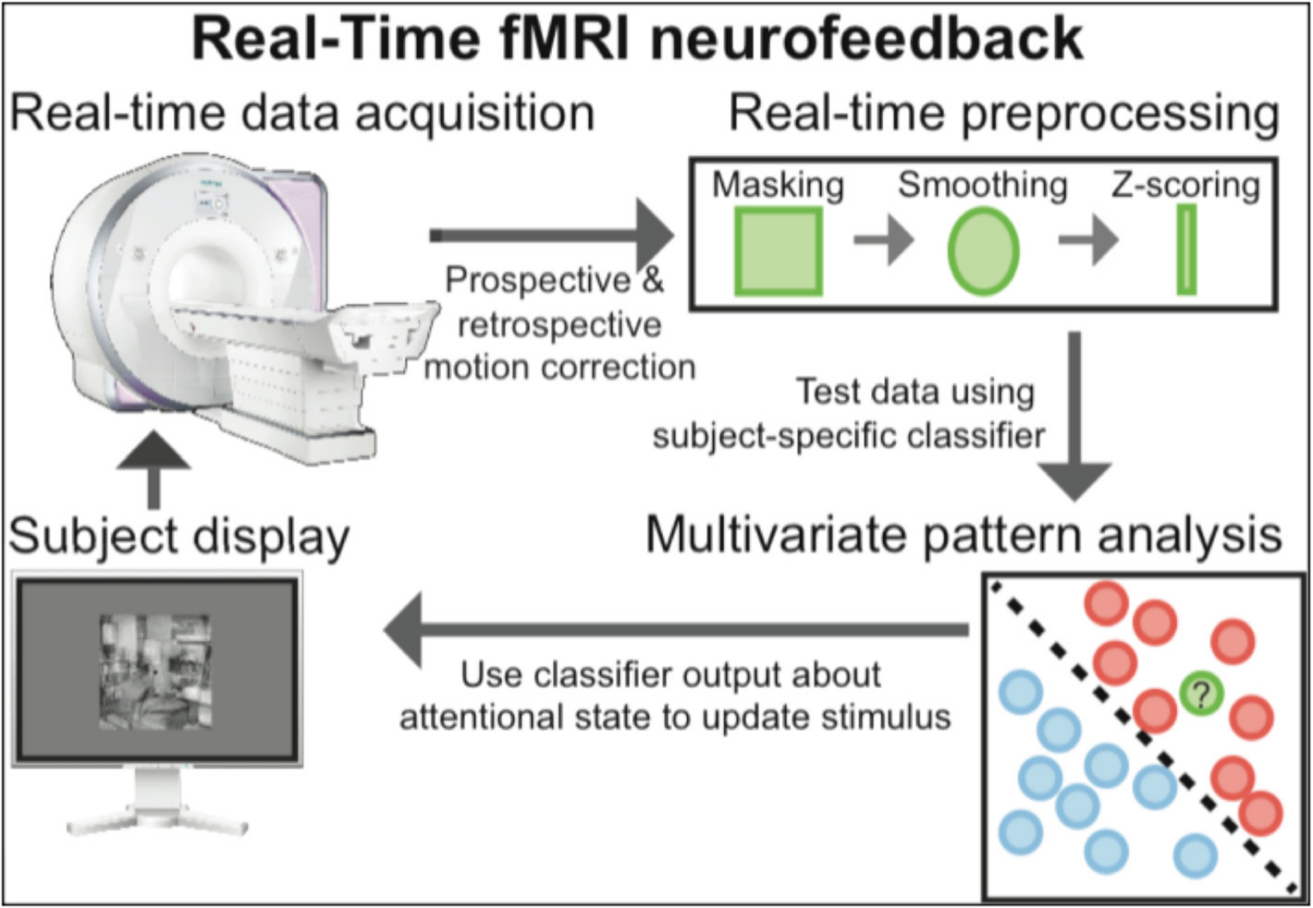
Overview of the real-time fMRI neurofeedback attention-training procedure. A video showing a typical visual display the participant might experience during the neurofeedback phase can be seen here - http://www.nature.com/neuro/journal/v18/n3/abs/nn.3940.html#videos. fMRI, functional magnetic resonance imaging.

We recently adapted a real-time fMRI neurofeedback approach developed for studying attention in the normal brain [17] to attempt to alter the neurobiology underlying the negative attention bias (Figure 1). In a pilot feasibility study, participants with elevated depression were trained to selectively attend to an emotionally neutral target category (for example, scenes) for a period of time while ignoring an emotionally salient distractor category (for example, sad faces). All experimental parameters were identical to those reported by deBettencourt, *et al*. [15], including scanner make and model and scanning and experimental protocols. Further, all procedures were approved by the Institutional Review Board at the University of Texas at Austin and participants provided written informed consent.

Each training session in this study involved a series of scanning runs in two phases: a classifier-training phase and a testing/feedback phase. During the training phase, fMRI data were collected from participants as they performed a task requiring selective attention to a continuous stream of composite images containing overlaid (neutral) face-and-scene stimuli. Participants alternated between attending to the face or scene while trying to detect rare lure images. These data were used to train a pattern classifier to decode neural activity that reflected attention to face *vs*. attention to scenes.

During the testing/feedback phase, fMRI data were collected and decoded in real time using the trained classifier. Participants were always instructed to attend to scenes, and sad faces were introduced as distractors. The output of the classifier provided evidence about whether participants were attending to the correct category (that is, scene), and this was translated (within 2 s) into feedback for the participant. Feedback took the form of altering the visual display to encourage correctly directed attention and discourage incorrectly directed attention. For example, while the participants were supposed to be attending to scenes, if the classifier indicated that sad faces were distracting them, the proportion of the scene stimulus in the composite image was *reduced* (for example, from 50% scene/face to 30% scene/70% face).

This feedback served to ‘externalize’ participants’ attentional state, making their distraction by the sad faces more tangible. This also made the task of attending to scenes more difficult, providing an error signal that distraction was undesirable. The logic was that participants could learn from this tangible feedback about good and bad attentional states and gain an ability to better monitor and control these states. The alternative approach of making the scenes more visible when distraction by the faces occurred might have helped participants in that moment to reorient to the scenes; however, this would potentially incentivize lapses. That is, to simplify the task demands in this regime, the best strategy would be to seek distraction rather than avoid it. Ultimately, the effectiveness of different feedback regimes awaits further empirical study, but the approach used here of making the task more difficult when attention lapsed has proven effective in controls [15] and in depressed individuals, as shown below.

**Figure 2:**
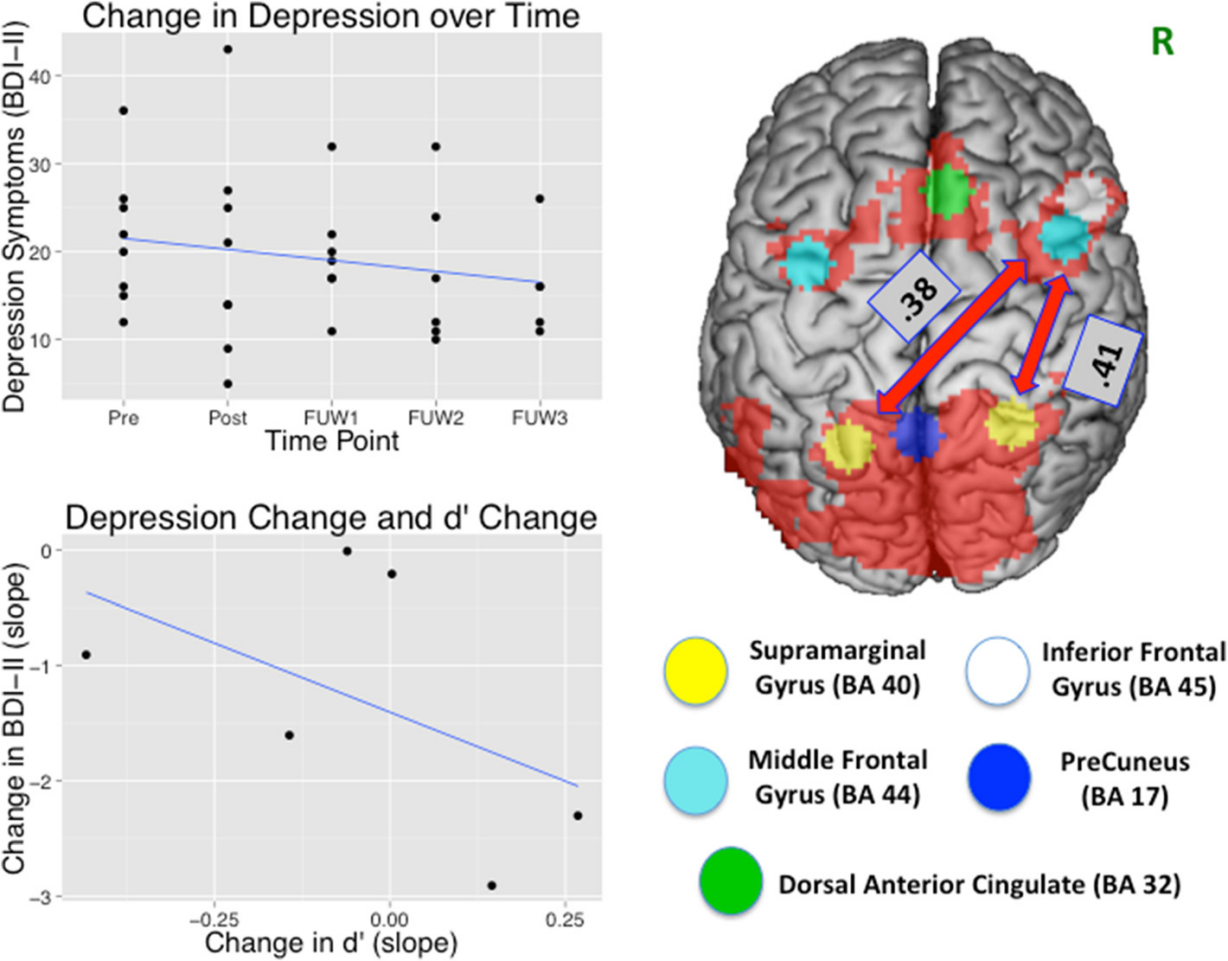
Preliminary results of feasibility study. Upper left panel graph shows BDI scores *pre* and *post* training and at three 1-week follow-ups (*FUW1*, *FUW2*, and *FUW3*). Lower left panel shows that changes in performance accuracy (indexed by d′ - a statistic that is calculated from hit and false alarm rates and hence reflects detection sensitivity) during training were associated with changes in BDI across this 4-week period. Mean d′ and standard deviation for performance across the 3 days were 1.06(.718), 1.32(.720), and 1.59(.871), respectively. The right panel shows the attention control network that was tested for pre-post changes in resting-state connectivity. This network was identified in previous work as associated with attention control and amenable to change with behavioral training [6]. All participants showed increased connectivity between right middle frontal gyrus (MFG) and bilateral supramarginal gyrus (SMG) of the parietal lobe. The mean and standard deviation in connectivity between right MFG and left SMG pre and post training were 0.11(0.18) and 0.38(0.26), respectively; between right MFG and right SMG 0.17(0.22) and 0.41(0.26), respectively. BA, Brodmann’s area; BDI, Beck Depression Inventory.

We ran a pilot study to demonstrate that this elaborate fMRI procedure is feasible in patients with depression. Seven adults with elevated symptoms of depression (mean Beck Depression Inventory-II [BDI-II] = 25; 4 female; mean age = 24) completed three sessions of neurofeedback training across a 5-day period, in between two laboratory assessment sessions. We were able to execute this procedure successfully, confirming the feasibility of the approach. Furthermore, the results were consistent with the possibility that this might be a useful approach. Specifically, improvements in attention control with training predicted improvements in mood symptoms across a 4-week follow-up period (Figure 2, left). Moreover, resting-state fMRI connectivity between frontal and parietal nodes of a previously identified attention control network [6] showed increased connectivity from before to after training (Figure 2, right).

These results must be interpreted with caution, as a control group was not included. Any future clinical study adopting this approach will need such a group, to ensure that the results cannot be attributed simply to practice with the task or other incidental aspects of the training. One control used in the previous study upon which this task was based [17] involved providing participants with sham feedback that was derived from other participants’ feedback sessions - and thus out of sync with their actual attentional state and thus presumably less useful for training. Future empirical work should include an appropriate active control condition.

## Conclusions

Neurocognitive therapeutics offers the promise of combining precision neural-monitoring techniques with behavioral training paradigms in order to increase the effectiveness of cognitive training. The critical difference between this approach and typical neurofeedback paradigms is that instead of directly presenting the individual with a measure of their brain activity, neurocognitive therapeutics uses that measure to dynamically alter the cognitive task itself. For attention training, real-time fMRI and multivariate analysis techniques can detect when attention is shifting and use that information to provide an error signal in the visual display being attended to help individuals learn to better control their attentional state. Although a long-term goal is to transition the neural-monitoring component from fMRI to a less costly, field-based technology, the initial use of fMRI is critical because it is currently the best technology for identifying distributed mental states non-invasively and with high fidelity. Our hope is that such translations of cutting-edge methods from cognitive neuroscience will increase the efficacy of cognitive training and clinical treatment.

## Abbreviations

BA: Brodmann’s area
BDI: Beck Depression Inventory
BOLD: blood-oxygen-level-dependent signal
d′: d prime
EEG: electroencephalography
fMRI: functional magnetic resonance imaging
MVPA: multivoxel pattern analysis
